# A multi spin echo pulse sequence with optimized excitation pulses and a 3D cone readout for hyperpolarized ^13^C imaging

**DOI:** 10.1002/mrm.28248

**Published:** 2020-03-15

**Authors:** Vencel Somai, Alan J. Wright, Maria Fala, Friederike Hesse, Kevin M. Brindle

**Affiliations:** ^1^ Cancer Research UK Cambridge Institute University of Cambridge Cambridge United Kingdom; ^2^ Department of Radiology, School of Clinical Medicine University of Cambridge Cambridge United Kingdom; ^3^ Department of Biochemistry University of Cambridge Cambridge United Kingdom

**Keywords:** hyperpolarized, imaging, lactate, pyruvate, tumor

## Abstract

**Purpose:**

Imaging tumor metabolism in vivo using hyperpolarized [1‐^13^C]pyruvate is a promising technique for detecting disease, monitoring disease progression, and assessing treatment response. However, the transient nature of the hyperpolarization and its depletion following excitation limits the available time for imaging. We describe here a single‐shot multi spin echo sequence, which improves on previously reported sequences, with a shorter readout time, isotropic point spread function (PSF), and better signal‐to‐noise ratio.

**Methods:**

The sequence uses numerically optimized spectrally selective excitation pulses set to the resonant frequencies of pyruvate and lactate and a hyperbolic secant adiabatic refocusing pulse, all applied in the absence of slice selection gradients. The excitation pulses were designed to be resistant to the effects of B_0_ and B_1_ field inhomogeneity. The gradient readout uses a 3D cone trajectory composed of 13 cones, all fully refocused and distributed among 7 spin echoes. The maximal gradient amplitude and slew rate were set to 4 G/cm and 20 G/cm/ms, respectively, to demonstrate the feasibility of clinical translation.

**Results:**

The pulse sequence gave an isotropic PSF of 2.8 mm. The excitation profiles of the optimized pulses closely matched simulations and a 46.10 ± 0.04% gain in image SNR was observed compared to a conventional Shinnar–Le Roux excitation pulse. The sequence was demonstrated with dynamic imaging of hyperpolarized [1‐^13^C]pyruvate and [1‐^13^C]lactate in vivo.

**Conclusion:**

The pulse sequence was capable of dynamic imaging of hyperpolarized ^13^C labeled metabolites in vivo with relatively high spatial and temporal resolution and immunity to system imperfections.

## 1 INTRODUCTION

Dynamic nuclear spin polarization of isotopically labeled metabolites has proven to be a promising technique for dynamic magnetic resonance imaging measurements of metabolism in vivo.^1^, ^2^ The principal limitation of the technique is the short lifetime of the spin hyperpolarization, which means that the signal is often observable for only a few minutes, requiring the use of very fast imaging methods.^3^, ^4^ [1‐^13^C]Pyruvate has been the most intensively investigated metabolite because of its central role in metabolism, the ease with which it can be hyperpolarized and its relatively fast membrane transport and subsequent metabolism when compared to the lifetime of the polarization.^5^ Hyperpolarized [1‐^13^C]pyruvate has been widely used in oncology, where imaging exchange of hyperpolarized ^13^C label between injected [1‐^13^C]pyruvate and the endogenous tumor lactate pool has been used to assess tumor grade,^6^ disease progression,^7^ and treatment response.^3^, ^8^ In general, increasing grade and progression are associated with an increase in lactate labeling and treatment response with a decrease. As well as being fast the imaging pulse sequence must also make economical use of the polarization because each excitation pulse results in depletion of the polarization, in addition to that due to *T*_1_‐dependent decay, degrading the signal‐to‐noise ratio (SNR) and decreasing the time window over which metabolism of the labelled substrate can be monitored. High‐resolution 3D volumetric imaging is desirable in order to fully assess tumor heterogeneity and treatment response, although the choice between a 2D or 3D k‐space acquisition strategy is not straightforward.^9^ Numerous pulse sequences have been proposed to address these issues (reviewed in^10^). Early studies used 2D chemical shift imaging (CSI) sequences,^11^, ^12^ which had relatively low spatial encoding efficiency, made uneconomical use of the hyperpolarization, and were not dynamically resolved. Multiband variable flip angle excitation pulses were introduced to better preserve the polarization. Larson et al used spectral‐spatial (SpSp) pulses, which can be used to selectively excite individual resonances in the slice of interest, for dynamic CSI measurements in vivo.^13^ These pulses have since been widely used in conjunction with a variety of different readout strategies. Lau et al imaged hyperpolarized ^13^C‐labeled pyruvate and bicarbonate in the heart with a rapid multislice sequence^14^ and recently used a simultaneous multislice sequence for accelerated imaging over a larger field of view (FOV).^15^ In the case of 3D acquisition strategies Miller et al introduced a 3D echo‐planar imaging sequence^16^ where the temporal resolution was traded for z‐resolution. In order to improve the temporal resolution of 3D acquisitions Wang et al recently proposed metabolite selective single‐shot spin echo‐based sequences^17^, ^18^ whereas Chen et al used a 3D pulse sequence with echo planar spectroscopic imaging (EPSI) readout and compressed‐sensing (CS).^19^ The advantage of imaging approaches where a specific resonance is excited selectively is that they are faster than EPSI. The disadvantage is that they require prior knowledge of the metabolite frequencies and are sensitive to nonuniform magnetic fields, which makes radiofrequency (RF) pulse design more challenging.^10^ Balanced steady state free precession imaging (bSSFP) can give higher SNR and efforts have been made to overcome the inherent frequency profile problems of this sequence.^20^, ^21^, ^22^, ^23^

An advantage of using SpSp pulses in the case of the hyperpolarized [1‐^13^C]pyruvate experiment is that a small flip angle pulse can be used to excite the intense pyruvate resonance, thus preserving polarization, while a larger flip angle pulse can be used on the less intense lactate resonance to improve SNR. A limitation of SpSp pulses is that the frequency profile of the pulses can lead to unwanted excitation in the stopband, resulting in cross‐contamination of the images and depletion of stored polarization. We have developed a single‐shot multi echo sequence with a 3D cone gradient readout, which avoids the need for a spatially selective excitation pulse when the imaging FOV exceeds the sensitive volume of the receiver coil. Such an approach, using a minimum phase, spectrally selective, and spatially nonselective RF pulse has been described previously.^24^ Relaxation of the requirement to be spatially selective enables a better optimized design of the pulse’s spectral profile and tolerance of B_1_ inhomogeneity. Although implemented on a high‐field (7T) preclinical scanner, the feasibility of implementing the sequence on a clinical scanner was demonstrated using lower gradient strengths and slew rates, even though this resulted in a much longer readout time than would be required clinically due to the smaller FOV and higher resolution in the preclinical experiment.

## 2 METHODS

### 2.1 Tumor model

EL4, murine lymphoma cells (American Type Culture Collection) were cultured in RPMI medium (Life Technologies, Carlsbad, California) containing 2 mM L‐glutamine and 10% fetal bovine serum (FBS) (Gibco/Thermo Fisher Scientific, Waltham, Massachusetts). Cells (viability >95%) were washed, resuspended in 0.2 ml phosphate‐buffered saline (PBS), and injected subcutaneously (5 × 10^6^) into the left flank of 8‐ to 12‐week‐old female C57BL/6J mice (Charles River Laboratories, Wilmington, Massachusetts). Tumors were allowed to develop for 10 days before imaging. All animal experiments were carried out in compliance with a project and personal licenses issued by the Home Office, UK and approved by the Cancer Research UK, Cambridge Institute Animal Welfare and Ethical Review Body.

### 2.2 Hyperpolarized [1‐^13^C]pyruvate

The sample contained 44 mg [1‐^13^C]pyruvic acid (CIL, MA), 15 mM OX063 (GE Healthcare, Amersham, UK), and 1.4 mM gadoterate meglumine (Dotarem; Guerbet, Roissy, France) and was hyperpolarized using a Hypersense Polarizer (Oxford Instruments, Abingdon, UK) at 1.2 K in a magnetic field of 3.35 Tesla (T), with microwave irradiation at 94.112 GHz. The sample was then rapidly dissolved in 6 mL buffer containing 40 mM HEPES, 94 mM NaOH, 30 mM NaCl 100 mg/L EDTA heated to 180 °C and pressurized to 10 bar.

### 2.3 MRI scanner

Experiments were performed at 7T (Agilent, Palo Alto, California). The maximum gradient strength in the pulse sequence was set to 4 G/cm and the slew rate to 20 G/cm/ms (maximum available values on the scanner: 40 G/cm and 200 G/cm/ms). A 42 mm diameter birdcage volume coil was used for ^1^H transmit and receive and a similar volume coil for ^13^C transmit. A 20 mm diameter surface coil was used for ^13^C receive (Rapid Biomedical GMBH, Rimpar, Germany).

### 2.4 Excitation pulse design

Optimized [1‐^13^C]lactate and [1‐^13^C]pyruvate excitation pulses were designed to give 45° and 5° flip angles respectively, in the passband without affecting the other three observed resonances. Slice selection was unnecessary because the 3D cone gradient readout gave a FOV that was larger than the sensitive volume of the surface coil receiver. Optimization employed a conventional Shinnar–Le Roux (SLR) design, which was then fine‐tuned using the built‐in fminsearch solver in MATLAB (The Mathworks, Natick, Massachusetts), which was used to adjust the amplitude and phase of the individual pulse points. The cost function (Equation 1) of the optimization consisted of four regions of interest, which were ±70 Hz (±0.93 ppm) of the lactate, pyruvate hydrate, and alanine resonances. Because pyruvate travels initially as a bolus throughout the body of the animals its signal can be far off‐resonance. Therefore, for pyruvate the region of interest was ±200 Hz (±2.65 ppm). The ideal frequency profile has the prescribed amplitude in the ±70 Hz passband around the lactate resonance for the lactate pulse and the ±200 Hz passband around the pyruvate resonance for the pyruvate pulse and zero in the other three spectral regions. A B_0_ field map acquired from an EL4 tumor using the same transmit and receiver coils is shown in reference.^18^ The potential variation in the lactate, pyruvate hydrate, alanine, and pyruvate resonance frequencies (less than ±15 Hz) resulting from B_0_ field inhomogeneity is well below the width of the spectral regions of interest. The width of these regions can also compensate for potential miscalibration of the ^13^C resonance frequency. Outside of these regions the pulse profile can be arbitrary. Furthermore, the pulse was designed to give constant phase in the passband immediately after the pulse in order to increase signal in the presence of large B_0_ and B_1_ field inhomogeneity and to minimize the achievable echo time. This is similar to a minimal phase or self‐refocusing SLR pulse.^25^ Deviation between the ideal and observed excitation profiles was quantified by means of residual sum of squares, evaluated in the four frequency bands of interest. To avoid too high pulse amplitudes a penalty term was added to the cost function to constrain the amplitudes to be less than 1.5 G

(1) $$Cost\;=W_{\mathrm{abs}}{∥\left|M_{\mathrm{ideal}}\right|-\left|M_{\mathrm{designed}}\right|∥}_{2}^{2}\;+\;W_{\mathrm{angle}}{∥∠M_{\mathrm{ideal}}-∠M_{\mathrm{designed}}∥}_{2}^{2}\;+\;W_{\mathrm{peak}}\left({∥RF\left(t\right)∥}_{\infty }>\text{max}B_{1}\right)$$

**FIGURE 1:**
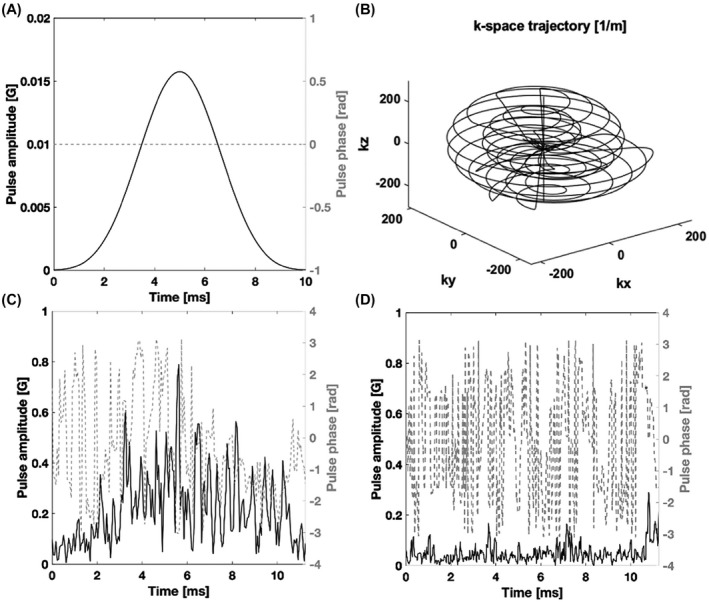
A, The amplitude (black line) and phase (gray dashed line) of the 10 ms linear phase Shinnar–Le Roux pulse. B, The 3D cones k‐space trajectory. C, The amplitude (black line) and phase (gray dashed line) of the optimized lactate pulse. D, The amplitude (black line) and phase (gray dashed line) of the optimized pyruvate pulse

**FIGURE 2:**
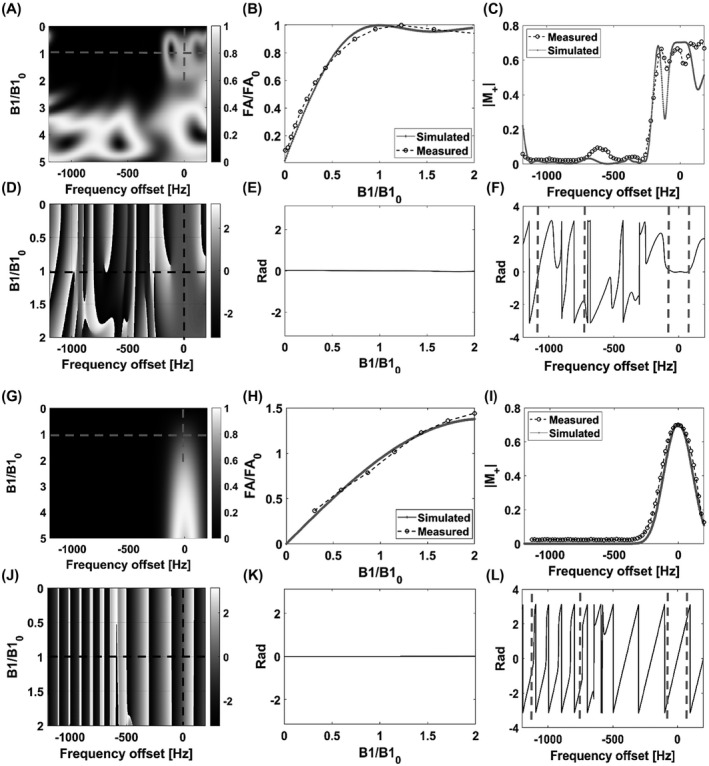
A, Theoretical transverse magnetization generated by the optimized lactate pulse for a range of B_1_ amplitudes relative to the nominal set value (*B*1_0_). The nominal set value is the peak amplitude, which for the lactate pulse was 0.79 G. The pulse was designed to give a 45° flip angle (FA_0_) at 0.5‐2 relative B_1_ amplitudes ±70 Hz of the lactate resonance frequency and a 0° flip angle ±70 Hz of the pyruvate‐hydrate and alanine resonances and ±200 Hz of the pyruvate resonance. B, Measured and theoretical flip‐angles relative to the design value (FA_0_) at 0 Hz frequency offset as a function of B_1_ amplitude relative to the nominal set value (*B*1_0_). Measurements were made on a 5 M thermally polarized [1‐^13^C]lactate phantom. C, Measured and theoretical transverse magnetization at the set nominal B_1_ value as a function of frequency offset. D, Theoretical signal phase profile for the optimized excitation pulse as a function of frequency offset and relative B_1_ amplitude. E, Theoretical signal phase at 0 Hz frequency offset as a function of relative B_1_ amplitude. F, Theoretical signal phase at the set nominal B_1_ amplitude plotted against frequency offset. The gray dashed lines indicate the spectral regions of interest for pyruvate (centered around −916 Hz) and lactate (centered around 0 Hz). G‐L, The corresponding plots for the standard SLR pulse

$M_{\mathrm{ideal}}$ and $M_{\mathrm{designed}}$ denote the desired and simulated frequency profiles, respectively. $W_{\mathrm{abs}}$ and $W_{\mathrm{angle}}$ represent weighted box functions for the four spectral regions of interest. $W_{\mathrm{abs}}$ represents the desired amplitude, while $W_{\mathrm{angle}}$ is the desired phase. The weights could be chosen to be higher for the pyruvate and lactate frequency bands, reflecting the greater importance of frequency selectivity for these resonances. However, this proved to be unnecessary and all the weights were set to unity in the optimization. $W_{\mathrm{peak}}$ is the penalty term if the maximum amplitude of the pulse exceeds $\text{max}B_{1}$. $W_{\mathrm{peak}}=10^{6}$ and $\text{max}B_{1}=1.5\;G$ were used in the design process. The resulting pulse performs similarly to an adiabatic SLR pulse^26^ but with better spectral selectivity for the design B_1_ amplitudes (from half to double the nominal B_1_ value) and lacks both the quadratic and linear phase variations that result from differences in frequency offset and B_1_ field strength, respectively. A further advantage of the pulse described here is that the spins are in phase at the end of the pulse, which facilitates echo timing. However, the pulse is less tolerant of B_1_ inhomogeneity than the adiabatic SLR pulse. The lactate and pyruvate pulse trajectories together with those for a linear phase SLR pulse are shown in Figure 1. The excitation profiles of the optimized lactate pulse and the corresponding SLR pulse are shown in Figure 2. To address B_1_ inhomogeneity the cost function was evaluated over an interval of pulse amplitudes (from half to double the nominal B_1_ value) and the final cost was the sum of the cost values at the sampled B_1_ values over this interval. Figure 2A shows that outside this B_1_ interval (0.5‐2) there is a loss of frequency selectivity, which is not the case for the SLR pulse (Figure 2G). This is an inevitable consequence of the numerical pulse optimization and means that the pulse needs to be redesigned if tolerance to B_1_ inhomogeneity over a different B_1_ interval is required. An additional constraint is imposed by the performance of the transmitter amplifier. Figure 1C,D show that the numerically optimized pulses require rapid changes in amplifier output. We found that it was important to include a smoothing function (we used the smooth() function in MATLAB with a span of 9) in each iteration of the numerical optimizer and evaluated the performance of the smoothed waveform in the cost function. Without smoothing we observed a greater discrepancy between the expected and observed pulse performance with increasing pulse power, which we assume reflects a failure of the amplifier to keep pace with requested changes in transmitter power.

The final optimized pulses were robust to both B_0_ and B_1_ inhomogeneity over the desired B_1_ interval, excite only the lactate or pyruvate resonances, and give high signal due to the constant phase in the passbands. The pulses were composed of 402 points, duration 11.256 ms, which is an integer multiple of the 4 μs sampling time in order to avoid interpolation. The peak B_1_ was 0.79 G for the lactate pulse and 0.29 G for the pyruvate pulse.

### 2.5 Pulse sequence

**FIGURE 3:**
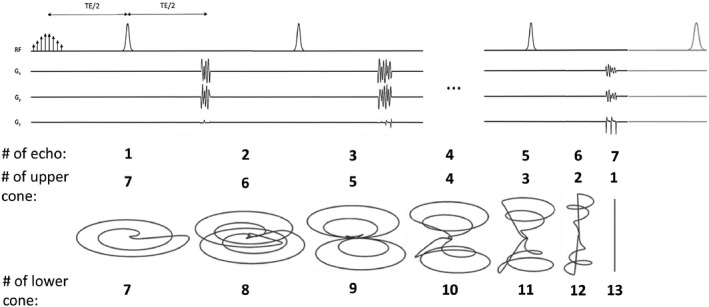
The pulse sequence starts with the optimized excitation pulse, without a slice selection gradient, and contains 7 unpaired adiabatic refocusing pulses to generate 7 spin echoes and an optional eighth adiabatic pulse at the end of the sequence that returns the remaining z magnetization back to the +z‐direction. At each echo a pair of two identical cones were acquired in decreasing order with respect to cone angle. The fine structure of the first cone is not visible due to the plot linewidth. The sequence takes 165 ms

The pulse sequence acquires a spherical k‐space (Figure 1) composed of 13 cone segments.^27^ each fully refocused with time‐optimal rewinders and distributed among 7 spin echoes. At each echo a pair of identical cones are sampled in decreasing order with respect to cone angle (ie, the 1st and 13th cones at the 7th echo, 2nd and 12th at the 6th echo etc.). The seventh cone is sampled twice at the first echo and the signal averaged to increase the SNR (Figure 3). The first cone of each pair is time reversed, going first to the edge of k‐space and then back to the center, whereas the second cone travels from the center to the edge so that the central part of k‐space is sampled at the peak of the echo. The first cone is similar to an in‐spiral readout and the second to an out‐spiral readout. The start point for the cones were rotated linearly around the symmetry axis (kz) so that an analytical density pre‐compensation could be used.^27^ The sampled k‐space was gridded to a 32 × 32 × 32 3D Cartesian grid using a Kaiser‐Bessel function with density post‐compensation. The final image was 16 × 16 × 16 resulting in a nominal isotropic resolution and FOV of 2 mm and 3.2 cm, respectively.

Adiabatic pulses with a hyperbolic secant profile were used to generate the spin echoes. The pulses were 10 ms long and designed to have a bandwidth of 10 kHz and to achieve >99.9% inversion over a frequency band of ±4 kHz at a B_1_ field strength between 1 to 64 Gauss. To avoid the quadratic phase imparted by these pulses no slice selection gradients were applied.^17^ In order to demonstrate the feasibility of clinical translation the gradient trajectory for the readout, which took 78.612 ms, was designed with maximal gradient amplitude and slew‐rate of 4 G/cm and 20 G/cm/ms, respectively. The k‐space trajectory measurement was performed according to.^28^

### 2.6 Phantom imaging

The pulse sequence was tested on a cylindrical phantom (7 mm inner‐diameter) filled with thermally polarized 5 M [1‐^13^C]lactate, placed in the magnet isocenter with the long axis of the phantom parallel with the z‐axis. The performance of the optimized pulse for lactate was compared to the original SLR design with respect to SNR. *T*_2_‐weighted proton images were acquired with a fast spin echo (FSE) sequence, to provide a positional reference, with a FOV of 32 mm, matrix size of 128 × 128 and 16 slices covering 32 mm in the z‐direction to maintain the same isotropic FOV as the ^13^C images.

### 2.7 Dynamic imaging in vivo

Interleaved pyruvate and lactate images were acquired for 60 s by alternately exciting and imaging the individual metabolites, with a repetition time of 1 s, resulting in a time resolution of 2 s per metabolite. Pyruvate image acquisition was initiated 10 s after the start of hyperpolarized [1‐^13^C]pyruvate injection (which finished 3‐5 seconds after the start of image acquisition) in order to allow sufficient time for conversion of some of the labeled pyruvate into lactate. Flip‐angles were calibrated on the lactate phantom. The carrier frequencies of the excitation pulses were calculated from the measured water proton frequency, based on prior measurements of the [1‐^13^C]lactate, [1‐^13^C]pyruvate and water proton resonance frequencies.^18^ The [1‐^13^C]pyruvate resonance frequency was at −916 Hz from the [1‐^13^C]lactate resonance frequency. *T*_2_‐weighted FSE proton images were acquired for positional reference with the same 32 mm isotropic FOV, 2 mm slice thickness and 0.25 mm in‐plane resolution. ^13^C images were reconstructed in MATLAB.

## 3 RESULTS

The frequency and amplitude profiles of the optimized lactate pulse were measured on a phantom containing 5 M thermally polarized [1‐^13^C]lactate and the results compared to simulated profiles (Figure 2A‐F), which were derived from Bloch‐simulated curves convolved with a Gaussian of 21 Hz full width at half maximum (derived from the width of the ^13^C lactate peak observed in vivo) to account for B_0_ inhomogeneity. Both the frequency and B_1_ profiles of the optimized pulse showed good agreement with the simulations. The pulse gave minimal excitation of the pyruvate and alanine resonances, although some pyruvate‐hydrate excitation might result from the transition band in the presence of large B_0_ inhomogeneity. The large phase variation for the stopband resonances will help to spoil signal arising from these resonances (Figure 2F). The corresponding profiles for the SLR pulse are shown in Figure 2G‐L. The optimized pulse showed constant phase in the passband (Figure 2F), as opposed to a steep linear phase variation for the SLR pulse (Figure 2L), and was much less sensitive to B_1_ inhomogeneity (compare Figure 2B,H). Similar results were obtained for the optimized pyruvate pulse.

**FIGURE 4:**
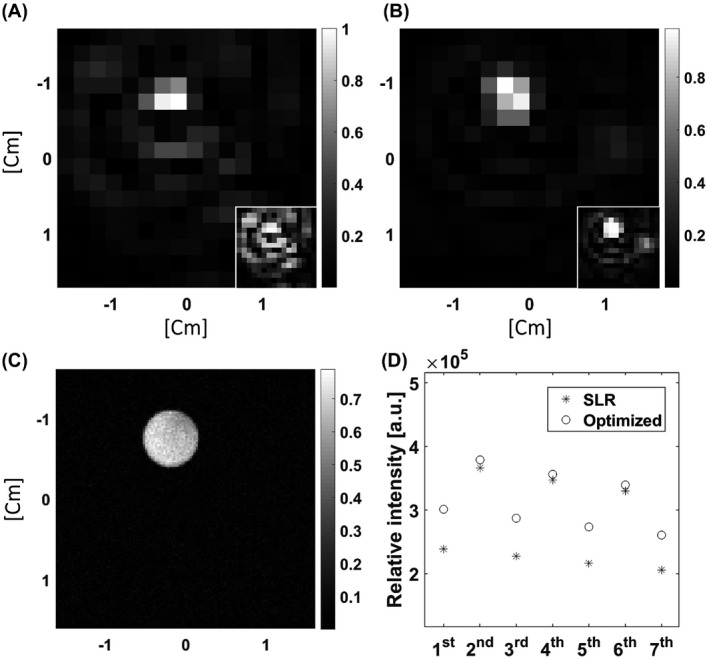
Axial ^13^C images from a cylindrical phantom containing 5 M thermally polarized [1‐^13^C]lactate. The shim values were set so that the linewidth was similar to that observed in vivo. A, Central slice of an image acquired with the Shinnar–Le Roux (SLR) excitation pulse. The same image is displayed in the inset with a decreased signal scale. B, The same slice acquired with the optimized excitation pulse. The same image is displayed in the inset with a decreased signal scale. C, Corresponding ^1^H image of the phantom. D, Echo amplitudes in the readout train acquired with the optimized lactate excitation pulse and the SLR pulse

**FIGURE 5:**
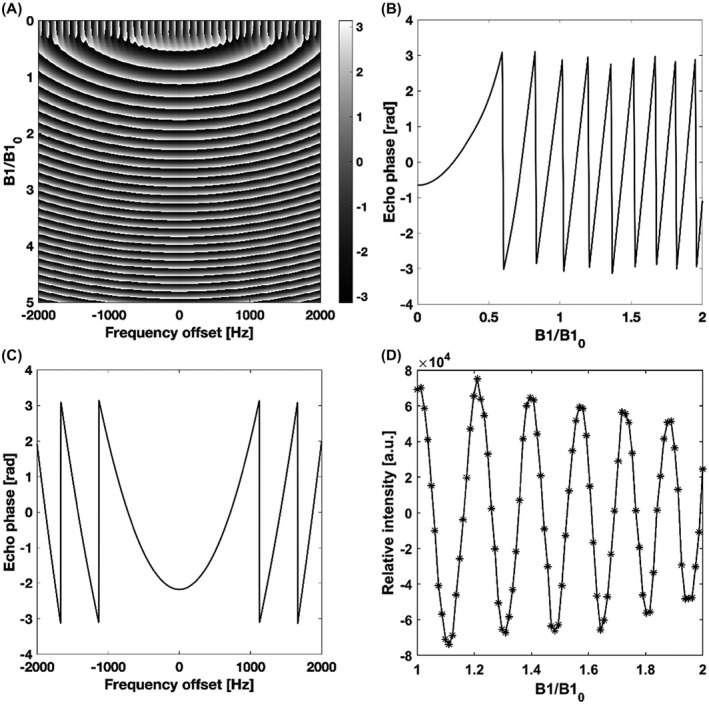
A, Simulated phase profile of the odd echoes as a function of frequency offset and relative B_1_ amplitude. B, The approximately linear phase ramp caused by varying the B_1_ field. C, Quadratic echo phase variation with frequency. D, Relative intensity of the real part of the first spin echo with increasing B_1_ amplitude

^13^C images were acquired from the same phantom using the SLR (Figure 4A) and optimized lactate excitation pulses (Figure 4B). The SNR for the central slice was 46.10 ± 0.04% (*n* = 20) higher for the optimized pulse, where this was defined as the ratio of the mean signal to the standard deviation of the image background. Image SNR can be degraded by the large B_1_ field‐dependent linear phase variation imparted by the adiabatic refocusing pulses (Figure 5). This will be further degraded by any phase variation left by the excitation pulse, especially if the matching of echo timing to the isodelay is imperfect, resulting in loss of signal at the odd echoes (the even echoes are fully refocused). The optimized pulse results in the spins being in phase at the end of the pulse, which is the case for the whole passband (Figure 2F), whereas for the SLR pulse the spins are in phase only at the center of the pulse and moreover this is strongly affected by frequency offset (Figure 2L). The decrease in amplitude of the odd echoes was less for the optimized pulse than for the SLR pulse (Figure 4D), which increases SNR and decreases image artifacts. This is illustrated by the insets in Figure 4A,B, which shows that the ring artifact associated with the point spread function (PSF) of the cone trajectory was less for the optimized pulse.

**FIGURE 6:**
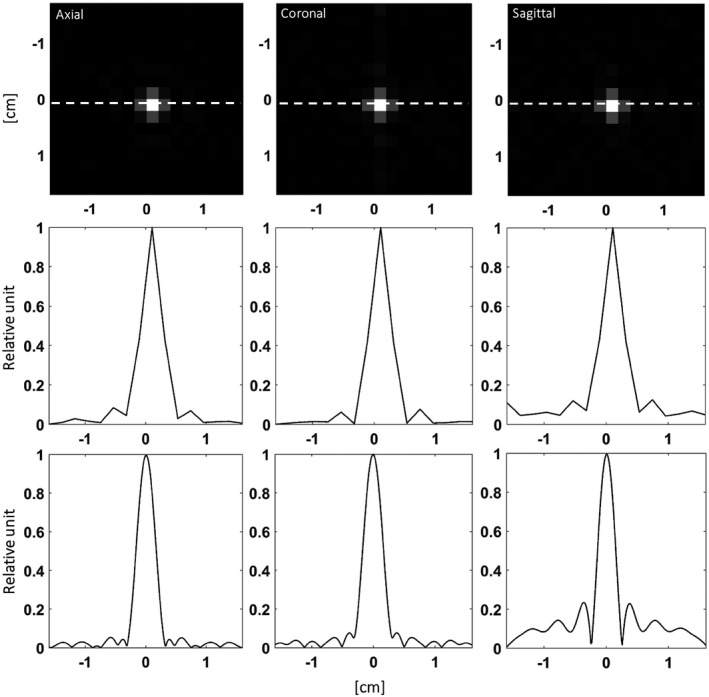
The central slices of the simulated point spread function (PSF) for the 3D cones readout reconstructed at the nominal 2 mm resolution. The numbers along the axes represent the distance from the isocenter in units of cm in each direction. The line profiles along the white dashed lines are plotted in the second row. The third row shows the same line profiles when the PSF was reconstructed at a digital resolution of 0.25 mm, which was the inplane resolution of the ^1^H images

The theoretical PSF of the 3D cones readout, ignoring relaxation, was simulated as described by Durst et al^29^ (Figure 6). A constant k‐space was sampled and 3D Fourier transformed after gridding to yield the PSF. The PSF was reconstructed at the digital resolution of the ^1^H images and gave theoretical profiles along the x, y, and z axes that were isotropic and had a full width at half maximum of 2.8 mm.

**FIGURE 7:**
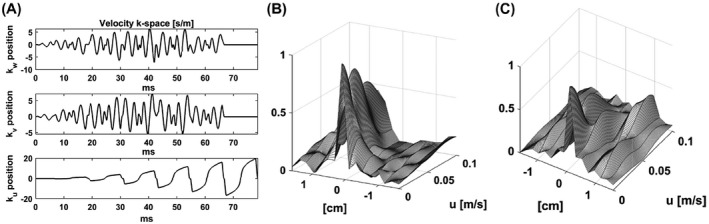
A, The velocity k‐space trajectory of the pulse sequence. B, The point spread function (PSF) z‐profile with respect to plug‐flow velocity along the z axis for the cone ordering shown in Figure 3. C, The PSF z‐profile with respect to plug‐flow velocity along the z axis with linear cone ordering (ie, first and second cones sampled at the first echo, third and fourth at the second echo, etc.)

The PSF will be affected by flow, particularly along the z‐axis where the spherical symmetry of the k‐space acquisition is broken by the echo train readout and in which direction the velocity of flow in vivo is expected to be greatest. To analyze the vulnerability of the sequence to flow, the effect of constant plug‐flow in the z‐direction was simulated. This type of flow induces a constant phase ramp along the direction of flow, which is proportional to the velocity of the spins (*u*) and their position in velocity k‐space (*k_u_* in the case of the z‐axis).^30^ The k‐space points accumulate a phase offset: $\Phi \left(k\left(t\right)\right)=\exp\left(-i2\pi uk_{u}\left(t\right)\right)$. Simulation of the PSF was carried out using a range of values for *u* $\left(u\in \left[0,\;u_{\text{max}}\right]\right)$. The maximal expected velocity was calculated to be 0.0615 m/s based on the cardiac output and aorta diameter of the mouse strain used in these experiments.^31^ The results of this simulation, up to a *u*_max_ of 0.1 m/s, compared to a simulation with a linearly sampled k‐space, that is, when consecutive cones are paired (1‐2, 3‐4, etc.) is shown in Figure 7. The readout is robust to flow up to the expected maximal velocity.

A similar analysis was used to assess the effect of relaxation. The individual cone‐pairs experience different *T*_2_ decay‐induced weighting, where the weighting of the *n*th echo can be approximated as $w\left(n\right)\;=\;e^{-bn}$; where *b* = $TE/T_{2}$. The first‐order change in the PSF z‐profile in the limit where there is no relaxation (*b* = 0) was determined both analytically and numerically. The Fourier‐transform of the projection of k‐space onto the *k_z_* axis yields the z‐axis PSF. The projection is equal to the area of the spherical cross‐section weighted by *T*_2_ relaxation‐induced exponential decay and the k‐space sampling density:

(2) $$P\left(k_{z}\right)=\int_{0}^{\sqrt{k_{R}^{2}-k_{z}^{2}}}2\cdot \pi \cdot k_{r}\cdot W\left(k_{r}\right)\cdot dk_{r}$$

(3) $$W\left(k_{r}\right)=1/\sqrt{k_{z}^{2}+k_{r}^{2}}\cdot 1/\cos\left(\text{atan}\left(k_{r}/k_{z}\right)\right)\cdot e^{-b*\text{atan}\left(k_{r}/k_{z}\right)}=1/k_{z}\cdot e^{-b*\text{atan}\left(k_{r}/k_{z}\right)}$$

*k_r_* denotes the radial integration variable and *k_z_* the position along the *k_z_* axis. Therefore, $k_{\theta }\;=\;\text{atan}\left(k_{r}/k_{z}\right)$. *k_R_* is the k‐space radius, therefore the upper bound of the integral at any given point along *k_z_* is given by $\sqrt{k_{R}^{2}\;-\;k_{z}^{2}}$. The additional factors, other than the exponential signal decay, represent an approximation of the k‐space sampling density, as described in,^27^ where *k_θ_* used here was denoted as *θ* in this earlier paper and a separate variable was used instead of $\sqrt{k_{R}^{2}\;-\;k_{z}^{2}}$ to express distance from the k‐space origin. The first derivative at *b* = 0 gives the change in the z‐profile of the PSF in the limit of no relaxation:

(4) $$\begin{array}{cc} {\left.\frac{\partial }{\partial b}{\text{PSF}}_{z}\right|}_{b=0}\; & =\;{\left.\frac{\partial }{\partial b}\left[F\left\{\int_{0}^{\sqrt{k_{R}^{2}-k_{z}^{2}}}2\;\cdot \;\pi \;\cdot \;k_{r}\;\cdot \;\frac{1}{\sqrt{k_{z}^{2}+k_{r}^{2}}}\;\cdot \;\frac{1}{\cos\left(\text{atan}\left(\frac{k_{r}}{k_{z}}\right)\right)}\;\cdot \;e^{-b*\text{atan}\left(\frac{k_{r}}{k_{z}}\right)}dk_{r}\right\}\right]\right|}_{b\;=\;0}\\ & =F\left\{-2\;\cdot \;\pi \;\cdot \;\frac{1}{2\;\cdot \;\left|k_{z}\right|}\;\cdot \;{\left[\left(k_{r}^{2}+k_{z}^{2}\right)\;\cdot \;\text{atan}\left(\frac{k_{r}}{k_{z}}\right)-k_{z}\;\cdot \;k_{r}\right]}_{k_{r}=0}^{k_{r}=\sqrt{k_{R}^{2}-k_{z}^{2}}}\right\}\\ & =F\left\{-\pi \;\cdot \;\frac{1}{\left|k_{z}\right|}\left(k_{R}\;\cdot \;\text{atan}\left(\frac{\sqrt{k_{R}^{2}-k_{z}^{2}}}{k_{z}}\right)-k_{z}\;\cdot \;\sqrt{k_{R}^{2}-k_{z}^{2}}\right)\right\} \end{array}$$

**FIGURE 8:**
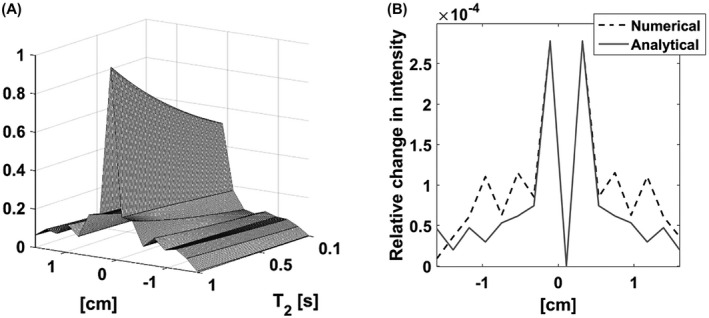
A, The effect of *T*_2_ relaxation time on the z‐profile point spread function (PSF). B, Comparison of the first‐order change in the z‐profile PSF at *R*_2_ = 0 derived analytically with the PSF z‐profile derived numerically from the difference between the profiles for *T*_2_ = 4s and for *R*_2_ = 0 (ie, when relaxation is neglected)

The inner integral was evaluated with Wolfram Alpha (Wolfram Research, Inc., Wolfram|Alpha Knowledgebase, Champaign, Illinois, 2019). The Fourier‐transform in Equation 4 was performed numerically. The theoretical PSF of the 3D cone trajectory, which included relaxation, was determined by numerical simulation, as described previously. Figure 8 shows the simulated PSF z‐profiles for different *b* values (Figure 8A) and the change in the profile at *b* = 0 derived by both simulation and analytically (Figure 8B).

Flow and *T*_2_‐relaxation had a negligible effect on the *x* and *y* profiles of the PSF and we have shown previously that despite the long echo train, with relatively long refocusing pulses, there is only a small increase in the rate hyperpolarization loss.^17^

**FIGURE 9:**
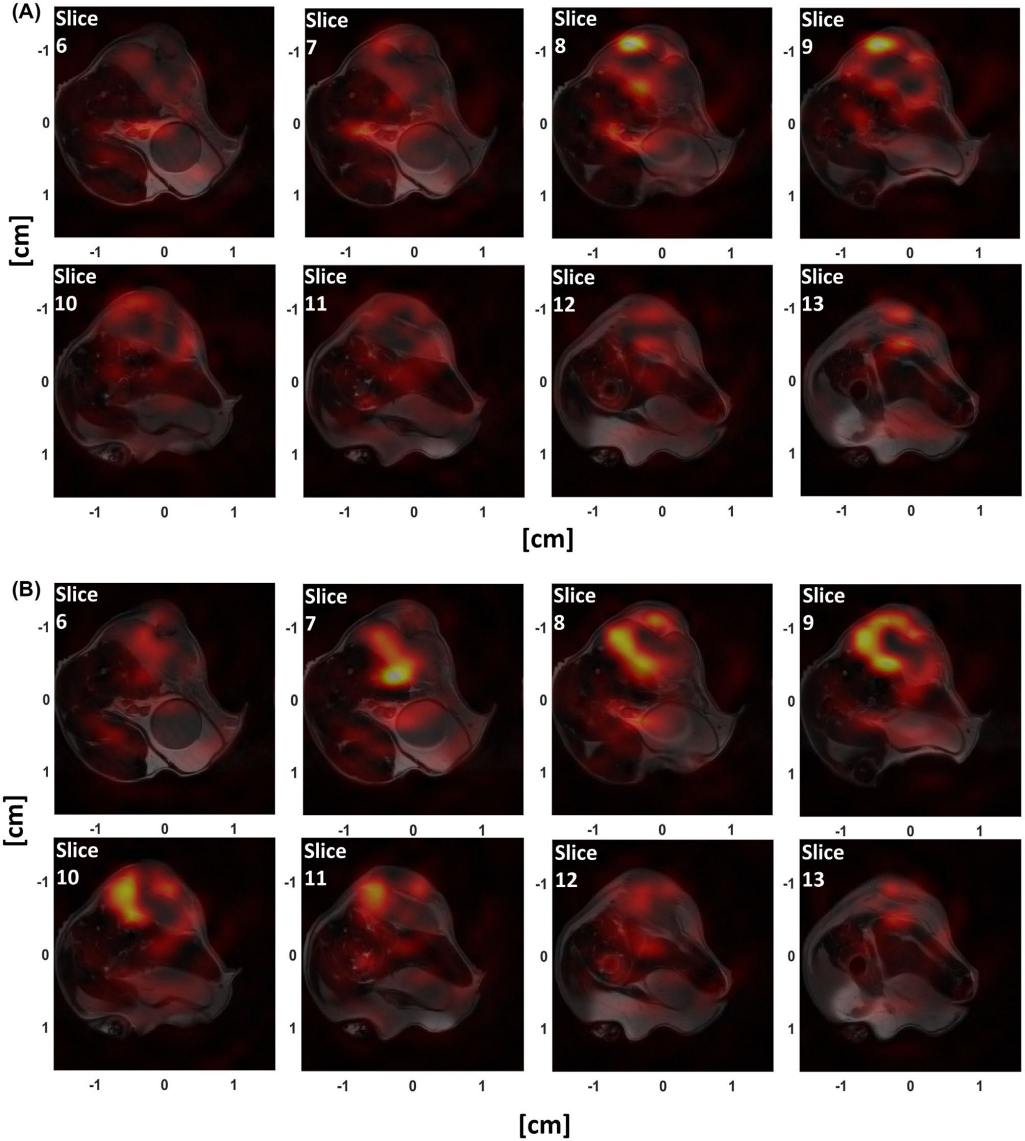
Hyperpolarized [1‐^13^C]pyruvate (A) and [1‐^13^C]lactate (B) images from a tumor‐bearing mouse overlaid on the corresponding ^1^H images (2 mm slice thickness). The ^13^C images were interpolated to the 128* ×* 128 in‐plane matrix size of the ^1^H image and summed in time over the first 20 s. The relatively small signal leakage from neighboring slices in slices 6 and 12 suggests that the simulated sampling point spread function profile is well preserved in vivo

**FIGURE 10:**
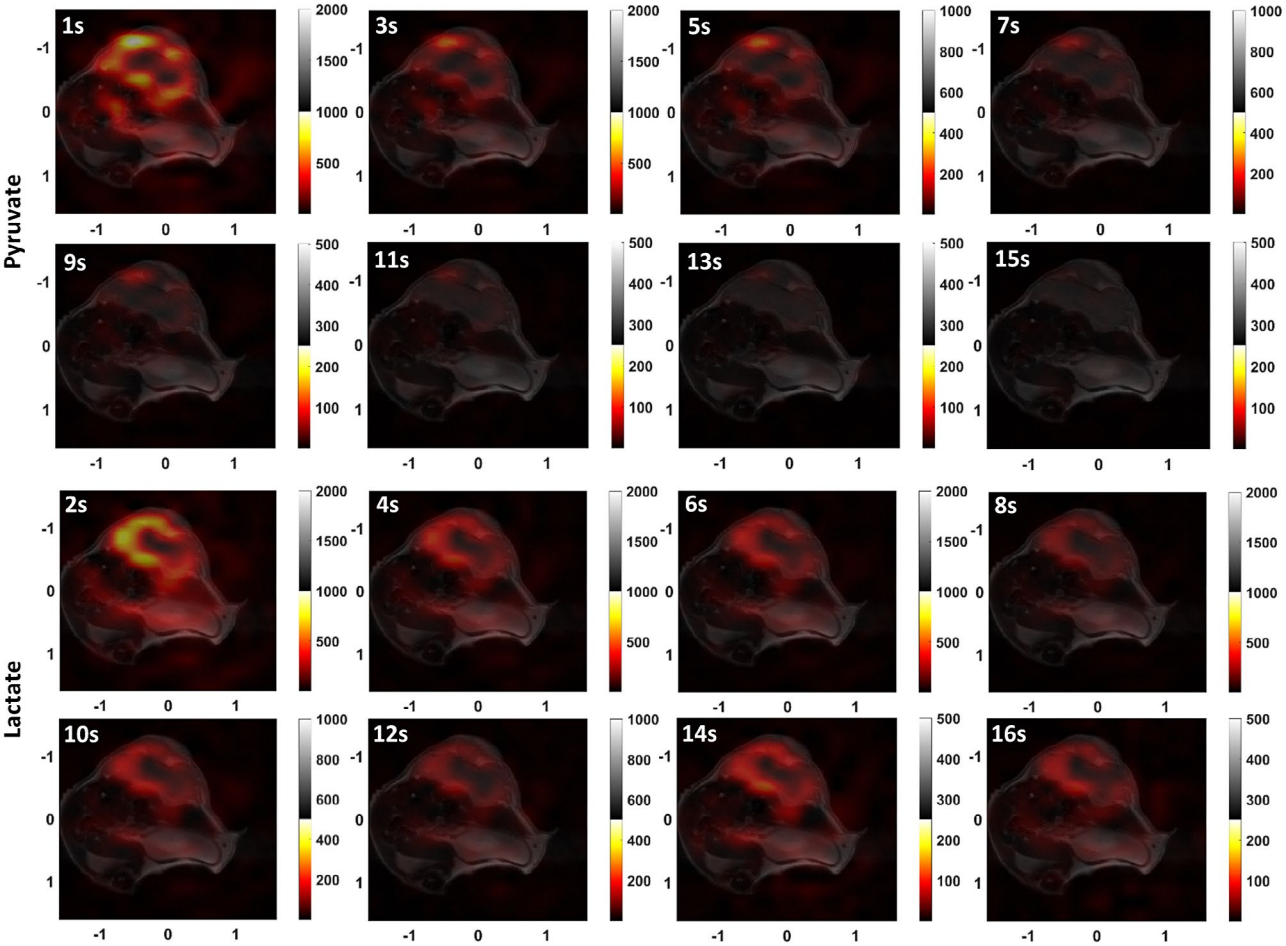
Dynamic hyperpolarized [1‐^13^C]pyruvate and [1‐^13^C]lactate images overlaid on the corresponding ^1^H image for slice 9. The odd time points correspond to pyruvate and the even time points to lactate. For better visualization of the ^13^C signal the scale is decreased at later time points

Dynamic hyperpolarized ^13^C images were acquired from a tumor‐bearing mouse. Figure 9 shows the [1‐^13^C]pyruvate and [1‐^13^C]lactate images obtained by summing images from the first 20 s of data acquisition overlaid on the corresponding *T*_2_‐weighted ^1^H images. The ^13^C images were interpolated to give the same 128 × 128 matrix size as the ^1^H images and were normalized to the highest intensity in the imaged volume. The acquisition started 10 s after the start of injection of hyperpolarized [1‐^13^C]pyruvate via a tail vein. Some pyruvate signal is detectable in the aorta in the seventh and eighth slices, but much higher intensity can be seen in the tumor region (Figure 9A), which shows a very similar distribution to the lactate signal (Figure 9B). Figure 10 shows individual images acquired from the central slice. The distribution of both metabolites was heterogeneous, as has been observed previously in this tumor model.^17^, ^18^

## 4 DISCUSSION

The pulse sequence allows 3D volumetric imaging after a single excitation, resulting in a short total readout time that minimizes vulnerability to B_0_ inhomogeneity and flow effects.^30^ When compared to 2D sequences the 3D readout benefits from fewer excitation pulses and the absence of slice cross‐talk, which helps to preserve the polarization. The sampling PSF is, to a good approximation, isotropic due to the spherical symmetry of the k‐space cone trajectory. The resolution observed in the phantom studies was similar to the simulated isotropic resolution of 2.8 mm. This could be degraded in vivo by $T_{2}^{*}$ relaxation, flow and field inhomogeneity effects. However, simulations showed these effects are small and in the studies on tumor‐bearing mice, the ^13^C signal did not exceed significantly the tumor volume in the z‐direction, which suggests that the PSF is well preserved in vivo.

The FOV of the 3D cone k‐space trajectory exceeded the sensitive volume of the receiver coil and therefore the excitation pulse did not need to be slice selective. Relaxation of the requirement to be slice selective facilitated optimization of the excitation pulse’s frequency profile, which decreased image degradation owing to the metabolite cross‐contamination that is often reported with similar sequences.^17^, ^18^, ^32^, ^33^ Contamination of the lactate image with pyruvate signal appears to be small as the background was correlated with the lactate signal rather than the pyruvate signal. The better phase characteristics of the optimized pulse, with a constant phase in the passband, as opposed to the steep linear phase variation for the standard SLR pulse, and its robustness to B_1_ inhomogeneity resulted in a smaller loss of signal at the odd echoes in the readout train, increasing image SNR and decreasing image artifacts. The lack of slice selection wastes some polarization if the excited volume exceeds the FOV, however this is balanced to some extent by the better spectral and phase profile of the optimized excitation pulse and the resultant increased image SNR and decreased image artifacts.

The sequence works with gradient strengths and slew rates similar to those used on clinical instruments, demonstrating the feasibility of clinical translation. With a 32 cm FOV and 2 cm resolution, typical of a clinical scanner, the sequence would take only 29.133 ms. The drawback of the sequence is that it contains a large number of adiabatic refocusing pulses and a long excitation pulse with relatively high B_1_ amplitudes, which may be an issue on a clinical scanner where specific absorption rate (SAR) is of primary concern. With a FOV of 16 cm and 0.5 cm nominal resolution, and the same 4 G/cm and 20 G/cm/ms gradient performance used here, the readout would take 136.4 ms and could be fit into four echoes. With an overdrive factor of 1.7 on the adiabatic and lactate excitation pulses the minimal repetition time on a clinical 3T MRI system (MR750, GE Healthcare, Waukesha, Wisconsin) is 2 s. Furthermore, the excitation pulse is expected to deposit less energy when stretched to cover the reduced spectral dispersion at the lower field. With a 32 cm FOV and 2 cm resolution these issues would be further ameliorated. The adiabatic pulses^34^ could also be optimized for (SAR), or alternatively balanced composite pulses could be used,^35^ which can outperform conventional adiabatic pulses with respect to compensation of both B_0_ and B_1_ field inhomogeneity.^34^ A (SAR) constraint could also be incorporated into the design of the excitation pulse. This would either mean an extra penalty term in addition to the pulse amplitude and constant passband phase terms, or an additional constraint if a constrained optimization solver is used in the design process. An additional and more significant problem with the hyperbolic secant refocusing pulses is that the refocused transverse magnetization accrues a B_1_‐dependent phase at the odd echoes. This results in signal distortion that can lead to artifacts, especially if very different parts of the k‐space are sampled at even and odd echoes. This problem could be addressed by a more symmetric segmentation of the 3D cones in k‐space. Another problem are edge effects, where the B_1_ field drops below the adiabatic threshold at the edge of the transmit coil leading to unwanted excitation and accelerated loss of polarization, which could be a particular problem on a clinical scanner. The feasibility of designing spectrally selective pulses for 3T has been demonstrated previously by Larson et al^13^ and Schulte et al,^33^ who used multiband and single resonance selective spatial‐spectral pulses, respectively, to image [1‐^13^C]labeled pyruvate and its downstream metabolites in vivo. For the optimized pulses described here, achieving the desired spectral bandwidth and sharp transition bands were the most difficult requirements to meet, whereas B_1_ robustness, reduction of ripples in the stopband and constant phase at the end of the pulse were more easily achieved. Therefore, we anticipate that starting the optimization process from pulses similar to those described in^13^ and^33^ will produce a pulse with the desired performance at 3T.

In conclusion, we have developed a 3D single‐shot multi spin echo sequence optimized for dynamic imaging of hyperpolarized [1‐^13^C]pyruvate and lactate. The sequence improves on that described in,^17^ with a smaller minimum repetition time, better SNR efficiency and an isotropic PSF that does not degrade the nominal resolution. The k‐space cone trajectory, with an FOV that exceeded the sensitive volume of the receiver coil, enabled the use of optimized excitation pulses that can increase SNR and reduce image artifacts. The feasibility of clinical translation was demonstrated with in vivo measurements at clinically relevant gradient strengths.

## CONFLICT OF INTEREST

KMB holds patents with GE Healthcare on some aspects of DNP technology.

## DATA AVAILBILITY STATEMENT

The code to design the k‐space readout and the numerically optimized excitation pulses is available at https://github.com/vs460/MultiSE.
